# Cytoprotective role of resveratrol in cigarette smoke-induced pyroptosis through Nrf2 pathway activation

**DOI:** 10.1016/j.cstres.2025.100107

**Published:** 2025-07-29

**Authors:** Mengyu Zhang, Chenyang Hu, Guang Yang, Yajie Hu, Yiqing Qu

**Affiliations:** Department of Pulmonary and Critical Care Medicine, Laboratory of Basic Medical Sciences, Qilu Hospital of Shandong University, Jinan, China

**Keywords:** Resveratrol, Chronic obstructive pulmonary disease, Nuclear factor erythroid 2-related factor 2, Pyroptosis, miR-200a

## Abstract

Resveratrol, a natural polyphenolic compound, has garnered increasing attention due to its antioxidant and anti-inflammatory properties. In this study, we investigated its protective role against cigarette smoke extract (CSE)-induced pyroptosis in human bronchial epithelial cell lines (BEAS-2B, 16HBE, and A549) and a chronic cigarette smoke (CS)-exposed mouse model. CS exposure is a major pathogenic factor in chronic obstructive pulmonary disease, primarily through promoting oxidative stress, inflammation, and pyroptotic cell death. Our results demonstrate that resveratrol enhances the activation of the nuclear factor erythroid 2-related factor 2 (Nrf2) signaling pathway, upregulating downstream antioxidant enzymes such as HO-1 and NQO1. This activation mitigates oxidative stress and inhibits the activation of the TXNIP/NLRP3/caspase-1 inflammasome axis. *In vitro*, resveratrol reduced ROS accumulation and proinflammatory cytokine release in CSE-stimulated human bronchial epithelial cells. *In vivo*, resveratrol partially restored lung function and redox homeostasis in CS-exposed mice. Moreover, mechanistic analyses revealed that resveratrol upregulates miR-200a expression, which directly targets Keap1, thereby relieving its inhibition of Nrf2. These findings suggest that resveratrol alleviates CSE-induced pyroptosis by modulating the miR-200a/Keap1/Nrf2 axis and may serve as a potential therapeutic strategy for smoking-related airway diseases. However, additional clinical studies are necessary to confirm its efficacy.

## Graphical abstract

**Figure:**
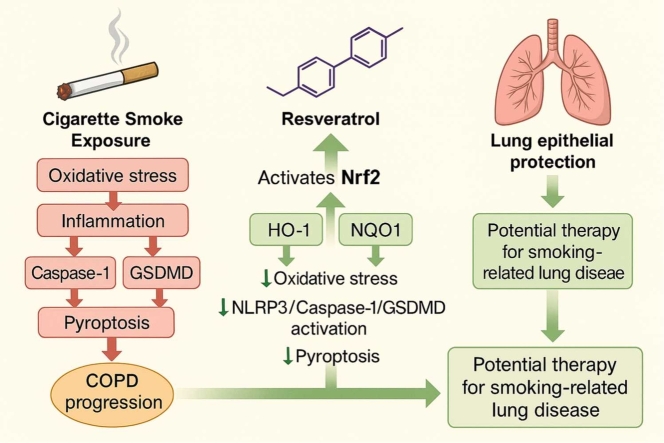

## Introduction

Chronic obstructive pulmonary disease (COPD) remains the third leading cause of disease-related death worldwide, imposing a substantial socioeconomic burden.^1^ Cigarette smoke (CS), the predominant etiological factor in COPD pathogenesis, has been experimentally demonstrated to induce pyroptotic cell death and activate inflammatory cascades in human bronchial epithelial cells *via* the NLRP3 inflammasome pathway. In our *in vitro* models, exposure to cigarette smoke extract (CSE) resulted in caspase-1 activation and interleukin-1β (IL-1β) secretion, thereby mechanistically linking oxidative stress to COPD progression through the canonical inflammasome pathway.^2^ Nevertheless, the precise molecular mechanisms underlying CS/CSE-induced pyroptosis remain incompletely understood.

Sustained increased reactive oxygen species (ROS) contributes to both airway and systemic inflammation, which are key pathological features in the development of COPD.^3^, ^4^ The overproduction of ROS, along with sustained activation of endogenous enzymes, resulted in direct damage to lung tissues exposed to CS.^5^, ^6^ Nuclear factor erythroid 2-related factor 2 (Nrf2) is a pivotal transcription factor involved in the cellular antioxidant response, while Kelch-like ECH-associated protein 1 (Keap1) binds to Nrf2 and promotes its degradation under basal conditions.^7^

The Keap1/Nrf2 antioxidant pathway has been implicated in the pathogenesis of various chronic inflammatory diseases.^8^, ^9^, ^10^ Studies have shown that Nrf2 knockout (Nrf2^-/-^) mice exposed to CS are more susceptible to severe emphysema and apoptosis, accompanied by significantly lower antioxidant enzyme activity compared to wild-type mice.^11^ These findings suggest that the Keap1/Nrf2 pathway plays a crucial role in modulating the progression of COPD. MicroRNAs (miRNAs) regulate gene expression by binding to the 3′-untranslated region (3′-UTR) of target mRNAs, thereby influencing protein synthesis.^12^ Among them, miR-200a has been identified as a potential regulator of Keap1 expression. Increased miR-200a levels have been shown to modulate the Keap1/Nrf2 pathway, improving the fructose-related hepatitis and lipid deposition and affecting liver fibrosis development.^13^, ^14^ Unfortunately, there is very little research related to miR-200a in COPD. Combined with pyroptosis and the research gap of miR-200a in COPD, we aimed to further explore the role of miR-200a.

Resveratrol (3,4’,5-trihydroxy-trans-stilbene) is a widely recognized polyphenolic stilbene compound present in grapes, mulberries, peanuts, rhubarb, and various other plants.^15^ Numerous studies have demonstrated that resveratrol possesses potent antioxidant, anti-inflammatory, and antitumor functions in many diseases.^16^, ^17^ In respiratory diseases, resveratrol also plays vital roles through antioxidant in lung injury caused by different substances.^18^ Resveratrol derivatives have been shown to modulate the Syk/NF-κB signaling pathway, thereby alleviating lipopolysaccharide-induced airway inflammation.^19^ Additionally, resveratrol attenuates lung inflammation and oxidative stress in COPD through the SIRT1/PGC-1α signaling pathway.^20^ However, it remains unclear whether resveratrol can modulate ROS levels through the Keap1/Nrf2 antioxidant pathway and the regulatory mechanism in COPD. The underlying regulatory mechanisms have not been fully elucidated. Therefore, the current study aimed to investigate whether resveratrol regulates pyroptosis in COPD through the Nrf2/Keap1 antioxidant signaling pathway.

## Materials and methods

### Preparation of CSE

CSE was prepared by bubbling smoke from two commercial cigarettes (11 mg tar, 0.9 mg nicotine, Taishan, Shandong) through 10 mL of prewarmed serum-free DMEM (Gibco) at a constant rate using a vacuum pump. The solution was filtered (0.22 µm, Millipore) and defined as 100% CSE when its absorbance reached 0.74 ± 0.05 at 320 nm. The extract was freshly prepared for each experiment and diluted to the desired concentration using DMEM (Gibco). In addition, CSE was used 30 min after preparation.

### Cell culture and treatment

Human bronchial epithelial cell lines BEAS-2B, 16HBE, and A549 (purchased from Procell Technology) were cultured in DMEM (Gibco) supplemented with 10% fetal bovine serum (Biological Industries) and 1% penicillin-streptomycin (Gibco) in a humidified incubator (37 °C, 5% CO_2_). Cells were seeded at a density of 1 × 10^5^ cells/cm^2^ and allowed to reach ∼80% confluence before treatment. CSE was prepared fresh daily and used at 5% concentration for 24 h in most experiments. Where indicated, cells were pretreated for 1 h with N-acetyl-L-cysteine (NAC, 5 mM, #A9165, Sigma), tBHQ (10 μM, #1948-33, Sigma), or resveratrol (20 μM, #1396, Selleck) before CSE exposure.

### ROS detection and oxidative stress assays

Intracellular ROS levels were measured using the DCFH-DA fluorescent probe (Beyotime, China, #S0035S) following the manufacturer’s protocol. Fluorescence intensity was quantified using spectro fluorophotometer (Shimazu, Japan, RF-6000). Superoxide dismutase (SOD) (#A001-2-1), malondialdehyde (MDA) (#A003-1-2), and GSH/GSSG levels (#A061-5-1) in cell lysates and bronchoalveolar lavage fluid were quantified using commercial assay kits from Nanjing Jiancheng Bioengineering Institute, following the manufacturer’s instructions.

### Western blotting

Total protein was extracted using RIPA lysis (Beyotime, China, #P0013C) buffer containing protease inhibitors. Equal amounts of protein (30 µg) were separated by sodium dodecyl sulfate polyacrylamide gel electrophoresis and transferred onto PVDF membranes. Membranes were blocked with 5% nonfat milk for 1 h and incubated overnight at 4 °C with the following primary antibodies:

**Table:** 

Target	Source	Catalog No.	Dilution
NLRP3	Abcam	ab210491	1:1000
Caspase-1	Abcam	ab179515	1:1000
GSDMD	Abcam	ab210070	1:1000
TXNIP	Abcam	ab188865	1:1000
Nrf2	Abcam	ab62352	1:1000
Keap1	Abcam	ab227828	1:1000
HO-1	Cell signaling technology	86806	1:1000
GST	Cell signaling technology	2622	1:1000
NQO1	Cell signaling technology	62262	1:1000

Abbreviations used: GSDMD, gasdermin D.

Secondary horseradish peroxidase-conjugated antibodies (1:5000) were applied for 1 h at room temperature, and bands were visualized using enhanced enhanced chemiluminescence Chemiluminescent Substrate Kit (#36222, Yeasen Biotechnology) and quantified using Image J.

### Quantitative reverse transcription-polymerase chain reaction

Total RNA was extracted from human bronchial epithelial cells subjected to different treatments, and its purity and concentration were assessed. The extracted RNA was then reverse transcribed into cDNA using the PrimeScript RT reagent kit (Takara). Finally, quantitative reverse transcription-polymerase chain reaction was performed using the TB Green Premix Ex Taq II kit (Takara). Glyceraldehyde 3-phosphate dehydrogenase (GAPDH) was used as the reference gene, and the relative gene expression levels were calculated using the 2^-ΔΔCT^ method.

All primers used in this study were designed and synthesized by Shanghai Biosharp Biotechnology Co, Ltd. The specific primer sequences used in this study are as follows:

**Table:** 

Target gene	Primer sequence (5′–3′)
NLRP3	Forward：GAGCCGAAGTGGGGTTCAGA Reverse：CTTCAATGCTGTCTTCCTGGC
caspase-1	Forward：TTGGAGACATCCCACAATGG Reverse：TGAAAATCGAACCTTGCGGA
GAPDH	Forward：AAATCAAGTGGGGCGATGCT Reverse：CAA ATGAGCCCCAGCCTTCT
TXNIP	Forward：GCCTCTGGGAACATCCTTCA Reverse：TCTCTTGAGTTGGCTGGCTC
Keap1	Forward：TCGTCTCCTTTATGCCGTGG Reverse：CATTCGCCACTCGTTCCTCT
Nrf2	Forward：CGCAGACATTCCCGTTTGTA Reverse：AGCAATGAAGACTGGGCTCT
ASC	Forward：GATCCAGGCCCCTCCTCAG Reverse：GCATCTTGCTTGGGTTGGTG

Abbreviations used: ASC, apoptosis-associated Speck-like protein containing a CARD; GAPDH, glyceraldehyde 3-phosphate dehydrogenase; Nrf2, nuclear factor erythroid 2-related factor 2 .

### Small interfering RNA (siRNA) transfection

SiRNAs including si-Nrf2, si-Keap1 and si-TXNIP was transfected into human bronchial epithelial cells using Lipofectamine 3000 reagent (Invitrogen). The transfection efficiency was evaluated at both the protein and mRNA levels. The siRNA sequences are as follows:

**Table:** 

siRNA	Sequences (5′–3′)
si-negative control (NC)	UUCUCCGAACGUGUCACGUTT
si-Nrf2-1	GGGAGGAGCUAUUAUCCAUTT
si-Nrf2-2	CCUGCUACUUUAAGCCAUUTT
si-Nrf2-3	CCUGAAAGCACAGCAGAAUTT
si-Keap1-1	CCUCAAUCGUCUCCUUUAUTT
si-Keap1-2	GGCGAAUGAUCACAGCAAUTT
si-Keap1-3	GUCCUGCACAACUGUAUCUTT
si-TXNIP-1	CAUCCUUCGAGUUGAAUAUTT
si-TXNIP-2	GUUGGGUAGAUCUGAACAUTT
si-TXNIP-3	GACAGCCCUAUCUUUAUGUTT

Abbreviation used: siRNA, small interfering RNA.

### Molecular docking analysis

We performed molecular docking analysis of the specific binding site between resveratrol and Keap1 using the GOLD program to obtain the most favorable docking conformations. After completing the molecular docking analysis, the optimal conformation of resveratrol was merged with the ligand-free Keap1 protein, and energy minimization was conducted using the Amber force field and Amber charges to form a new ligand-protein complex. Finally, the ligand-protein complex was further optimized and visualized using PyMol software.

### Dual-luciferase reporter assay

The 3′-UTR sequence of the human Keap1 domain was inserted into the GP-miRGL0 control vector, and the primers used for the Keap1 3′-UTR sequence are listed in Figure. The day before the experiment, 16HBE, BEAS-2B, and A549 cells were seeded into 24-well plates, with 500 μL of cell suspension per well. The experiment was divided into different groups, and all cells were collected. The Dual-Luciferase Reporter Assay Kit was used to measure luciferase activity in different groups to determine whether miR-200a can bind to Keap1 and identify the specific binding site.

### Animal model and ethics

Male C57BL/6J mice (7 weeks old, 18–20 g) were obtained from the Experimental Animal Center of Shandong University. The CS-induced COPD mouse model was established by exposing mice to the smoke of 20 cigarettes per day, 5 days per week, for 6 months. The resveratrol group received intratracheal instillation of resveratrol (20 mg/kg) 30 min before each smoke exposure. All animal experimental procedures were approved by the Laboratory Animal Ethics and Welfare Committee of Shandong University with Approval No. KYLL-2022 (ZM)-1062, and all methods were performed in accordance with the relevant guidelines and regulations, including the ARRIVE and NIH guidelines. Mice were euthanized using intraperitoneal injection of pentobarbital sodium (100 mg/kg) in accordance with institutional and national ethical standards.

### Pulmonary function measurement of animals

Whole-body plethysmography (WBP) technology was used for the pulmonary function measurement of animals. Compared with traditional plethysmography, WBP technology avoids the need for animal restraint or anesthesia, allowing for repeated measurements. These key features make WBP a suitable method for longitudinal studies, enabling the evaluation of progressive respiratory changes under physiological and pathological conditions. Compared to traditional plethysmography techniques, assessing respiratory function through WBP demonstrates better experimental reproducibility and is more convincing. Additionally, plethysmography is a well-established technique for measuring human lung function. Once all equipment was properly connected, pulmonary function in the mice was assessed using the FlexiVent system. The pulmonary function indexes, including Ti, Te, Rpef, end-inspiratory pause (ms), end-expiratory pause (ms), TVb (mL), Penh, pause, EF50 (mL/s), Tr (ms), peak inspiratory flow (mL/s), PEF (mL/s), F (beats per minute), and MVb (mL),were registered.

### Histological examination

Histological analysis and immunohistochemical staining were performed following standard protocols.

### Enzyme-linked immunosorbent assay

The human bronchial epithelial cells were stimulated with CSE for 12 h. Proteins were extracted, and the supernatants were collected for further analysis. All the inflammatory cytokines were quantified in the culture supernatants using Enzyme-linked immunosorbent assay kits (Elabscience) according to the manufacturer’s instruction.

### Statistical analysis

All data analyses in this study were performed using IBM SPSS Statistics version 24 (IBM Corp., Armonk, NY, USA) and GraphPad Prism version 8 (GraphPad Software, Dotmatics, San Diego, CA, USA). Continuous quantitative data were presented as mean±standard deviation (mean±SD) or as median with interquartile range. Differences between the control and experimental groups were analyzed using Student’s t-test. For statistical comparisons among three or more groups, one-way analysis of variance was performed, followed by Tukey’s multiple comparison test. *P* < 0.05 was considered statistically significant.

## Results

### CSE exposure induces oxidative stress and activates the NLRP3 inflammasome in human bronchial epithelial cells

**Fig. 1:**
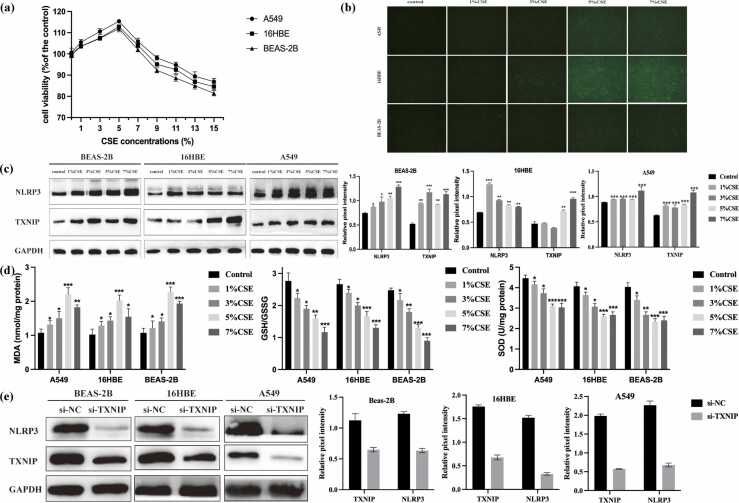
CSE causes oxidative stress and activates NLRP3 inflammasome in human lung epithelial cells. (a) CCK-8 assay was performed to explore the effect of CSE with different concentrations (1%, 3%, 5%, and 7%). (b) After different doses of CSE (1%, 3%, 5%, and 7%) exposed for 24 h in A549, 16HBE, and BEAS-2B cells, total ROS was measured. (c) Western blot analysis showed the expression levels of TXNIP and NLRP3 in dose-depend manner. (d) After treated with different concentrations of CSE (1%, 3%, 5%, and 7%) for 24 h in A549, 16HBE, and BEAS-2B cells, the expression levels of oxidants (MDA) and antioxidants (GSH/GSSG and SOD) were determined. (e) Western blot analysis showed the expression levels of NLRP3 and TXNIP after si-TXNIP transfection. The data are expressed as the mean of 3 independent experiments±standard deviation, *P < 0.05, **P < 0.01, and ***P < 0.001 *versus* the control group. Abbreviations used: CSE, cigarette smoke extract; ROS, reactive oxygen species; HBE, human bronchial epithelial; NS, not significant.

To evaluate the cytotoxic range of CSE, BEAS-2B, 16HBE, and A549 cells were treated with increasing concentrations (1–10%) for 24 h. Concentrations ≤7% did not cause significant cytotoxicity, and 5% was selected for subsequent experiments (Figure 1(a)). Exposure to CSE significantly increased intracellular ROS levels in a dose-dependent manner, indicating disrupted redox homeostasis (Figure 1(b)). Western blot analysis showed that CSE treatment led to increased expression of TXNIP and NLRP3, suggesting that CSE activates the NLRP3 inflammasome (Figure 1(c)). Additionally, CSE-treated cells exhibited elevated MDA levels and reduced antioxidant activity, as reflected by decreased GSH/GSSG ratio and SOD levels (Figure 1(d)). To further investigate the role of TXNIP, cells were transfected with si-TXNIP. The result showed si-TXNIP notably reduced the expression levels of TXNIP and was accompanied by a decrease in NLRP3 protein levels (Figure 1(e)), suggesting a possible regulatory link between TXNIP and NLRP3 expression that warrants further mechanistic investigation.

### CSE triggers pyroptosis and proinflammatory cytokine release

**Fig. 2:**
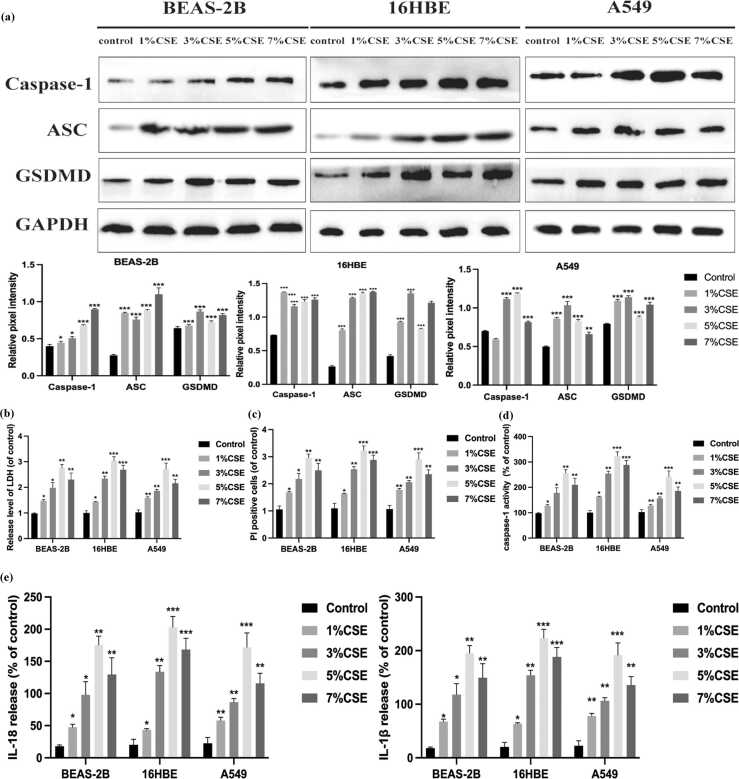
CSE promotes pyroptosis and inflammation in human lung epithelial cells. (a) Western blot analysis showed the expression levels of Caspase-1, ASC, and GSDMD after different dose of CSE (1%, 3%, 5%, and 7%) exposed for 24 h in A549, 16HBE, and BEAS-2B cells. (b) LDH release level were measured in A549, 16HBE, and BEAS-2B cells with different concentrations (1%, 3%, 5%, and 7%). (c) Analysis of the number of PI-positive cells in different concentrations of CSE-treated A549, 16HBE, and BEAS-2B (1%, 3%, 5%, and 7%). (d) CSE-increased caspase-1 activity in A549, 16HBE, and BEAS-2B cells. (e) CSE enhanced the release level of IL-1β and IL-18 in A549, 16HBE, and BEAS-2B cells.The data are expressed as the mean of three independent experiments±standard deviation, *P < 0.05, **P < 0.01, and ***P < 0.001 *versus* control group. Abbreviations used: CSE, cigarette smoke extract; GSDMD, gasdermin D; LDH, lactate dehydrogenase; IL, interleukin; PI, propidium iodide; HBE, human bronchial epithelial.

Following CSE stimulation, we observed a dose-dependent increase in the expression of pyroptosis-associated markers, including caspase-1, ASC, gasdermin D, IL-1β, and IL-18 (Figure 2(a)). LDH release and propidium iodide-positive staining confirmed increased membrane damage in CSE-treated cells (Figure 2(b) and (c)). Caspase-1 enzymatic activity and secretion of IL-1β and IL-18 were significantly elevated after CSE exposure, indicating robust pyroptotic response and inflammatory activation (Figure 2(d) and (e)).

### CSE disrupts the Keap1/Nrf2 antioxidant pathway

**Fig. 3:**
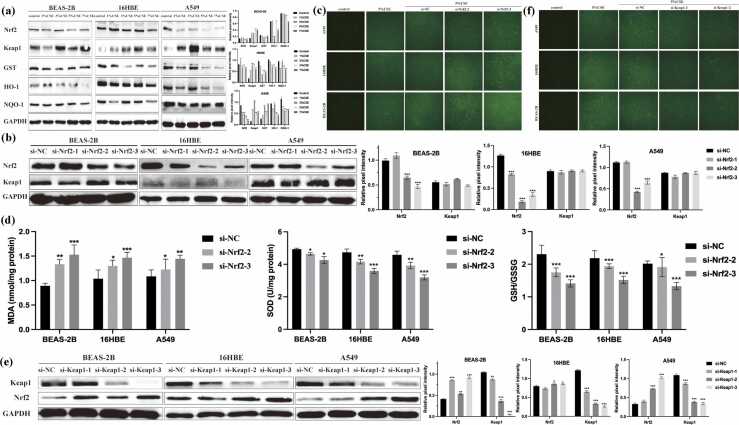
Evaluation of CSE on Keap1/Nrf2 pathway and oxidative stress in human lung epithelial cells. (a) Western blot analysis showed the expression levels of Nrf2, Keap1, GST, HO-1, and NQO1 in A549, 16HBE, and BEAS-2B cells with different concentrations CSE (1%, 3%, 5%, and 7%) treated. (b) Western blot analysis showed the expression levels of Nrf2 and Keap1 after si-Nrf2 transfection. (c) After different doses of CSE exposed for 24 h, A549, 16HBE, and BEAS-2B cells were transfected with si-Nrf2 and total ROS were measured. (d) After treated with CSE for 24 h, A549, 16HBE, and BEAS-2B cells were transfected with si-Nrf2 and the expression levels of oxidants (MDA) and antioxidants (GSH/GSSG and SOD) were determined. (e) Western blot analysis showed the expression levels of Nrf2 and Keap1 after si-Keap1 transfection. The data are expressed as the mean of 3 independent experiments ± standard deviation, *P < 0.05, **P < 0.01, and ***P < 0.001 *versus* control group. Abbreviations used: CSE, cigarette smoke extract; LDH, lactate dehydrogenase; HBE, human bronchial epithelial.

In previous experiments, we identified that CSE concentrations exceeding 7% caused significant cytotoxicity. Based on these results, we chose 5% CSE as the optimal concentration for subsequent experiments. We next examined the impact of CSE on the Keap1/Nrf2 signaling axis. Western blot revealed that CSE treatment led to decreased Nrf2 and downstream antioxidant proteins HO-1, GST, and NQO1, while increasing Keap1 protein levels (Figure 3(a)). To assess the functional role of Nrf2, we knocked down Nrf2 using siRNA. Effective silencing of Nrf2 enhanced ROS production and further altered redox biomarkers (MDA, SOD, GSH/GSSG) (Figure 3(b)-(d)). Interestingly, Keap1 silencing *via* siRNA restored Nrf2 expression and reversed oxidative stress, supporting its negative regulatory role (Figure 3(e) and (f)). These findings suggest that CSE exposure may contribute to oxidative stress in human lung epithelial cells, potentially through disruption of the Keap1/Nrf2 pathway, as indicated by altered expression of antioxidant proteins. Together, these results suggest that CSE induces oxidative injury partly by disrupting the Keap1/Nrf2 redox balance.

### Discovery of resveratrol as an Nrf2 activator

**Fig. 4:**
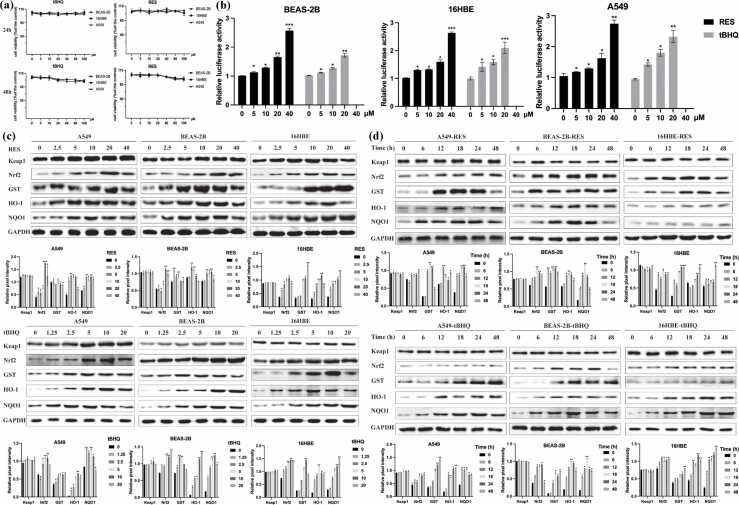
Identification of resveratrol as Nrf2 activators in human lung epithelial cells. (a) BEAS-2B, 16HBE, and A549 cells were treated with different doses of RES and tBHQ for 24 h and 48 h, and then cell viability was examined using the CCK-8 assay. (b) RES and tBHQ induced ARE-luciferase activity in a dose-dependent manner. BEAS-2B, 16HBE, and A549 cells were transfected with ARE-luciferase plasmid and renilla luciferase expression plasmid, and then treated by different concentrations of RES and tBHQ for 24 h. (c) RES and tBHQ dose-dependently induced the protein expressions of Keap1, Nrf2, GST, HO-1 and NQO1, but had no effect on Keap1. Cells were treated by indicated doses of RES and tBHQ for 24 h, and then total cell lysates were determined by immunoblot analysis. (d) RES and tBHQ induced Nrf2, GST, HO-1, and NQO1, but had no effect on Keap1 in the time-course study. The data are expressed as the mean of 3 independent experiments±standard deviation, *P < 0.05, **P < 0.01, and ***P < 0.001 *versus* control group. Abbreviations used: CSE, cigarette smoke extract; LDH, lactate dehydrogenase; HBE, human bronchial epithelial; RES, resveratrol.

Building upon previous studies suggesting that tBHQ (a known Nrf2 activator) offers protective effects in various models, we hypothesized that resveratrol could also activate the Nrf2 pathway in human bronchial epithelial cells. To verify this, we first evaluated the cytotoxicity of tBHQ and resveratrol using CCK-8 assays on BEAS-2B, 16HBE, and A549 cells. Figure 4(a) demonstrates that neither tBHQ (up to 20 μM) nor resveratrol (up to 40 μM) exhibited cytotoxicity after 48 h of exposure. Both compounds were found to induce Nrf2 transcriptional activity in a dose-dependent manner, with resveratrol achieving the highest induction at 40 μM (Figure 4(b)). Protein expression analysis revealed that resveratrol elevated the Nrf2 and its downstream targets, such as NQO1, GST, and HO-1, in a concentration-dependent manner (Figure 4(c)). Notably, treatment with tBHQ and resveratrol did not affect Keap1 expression. Time-course analysis showed that Nrf2 activation peaked at 24 h, followed by a gradual decline (Figure 4(d)). These results confirm that resveratrol activates the Nrf2 pathway and upregulates cytoprotective genes in human bronchial epithelial cells.

### Resveratrol attenuates CSE-induced oxidative stress and pyroptosis

**Fig. 5:**
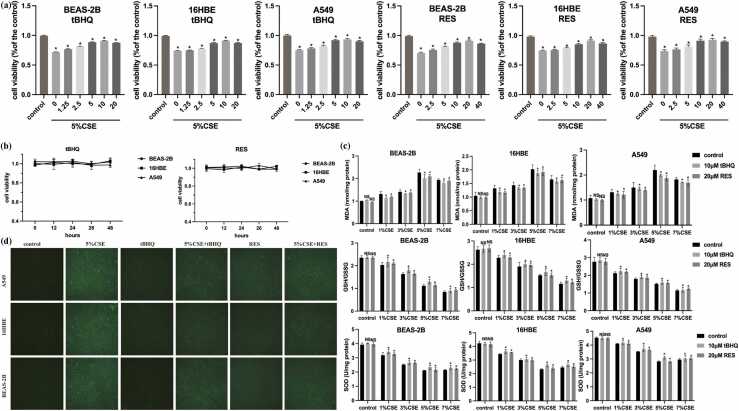
Resveratrol protect human lung epithelial cells against CSE-induced oxidative stress. (a) RES and tBHQ protected BEAS-2B, 16HBE, and A549 cells against CSE-induced cell death, and cell viability was measured using the CCK-8 assay. (b) Cell cytotoxicity in BEAS-2B, 16HBE, and A549 cells treated with 10 μM tBHQ and 20 μM RES alone without any cytotoxicity. (c) RES and tBHQ dose-dependently reduced the CSE-increased MDA level and increased the CSE-reduced GSH/GSSG and SOD level. (d) RES and tBHQ reduced the 5%CSE-induced upregulation of intracellular ROS level. BEAS-2B, 16HBE, and A549 cells were pretreated with tBHQ (10 μM) and RES (20 μM) for 24 h. The level of ROS was determined using the ROS detection kits. The data are expressed as the mean of 3 independent experiments±standard deviation, *P < 0.05, **P < 0.01, and ***P < 0.001 *versus* control group. Abbreviations used: CSE, cigarette smoke extract; HBE, human bronchial epithelial; RES, resveratrol.

In this part, we first assessed the protective effects of resveratrol against CSE-induced oxidative stress in human bronchial epithelial cells. Pretreatment with resveratrol significantly reduced ROS accumulation and reversed CSE-induced changes in MDA, SOD, and GSH/GSSG levels. Figure 5(a) shows that pretreatment with 20 μM resveratrol reduced CSE-induced cell death. Most importantly, exposure to 10 μM tBHQ and 20 μM resveratrol alone did not exhibit any cytotoxic effects (Figure 5(b)). Moreover, resveratrol significantly mitigated CSE-induced oxidative stress, as evidenced by restored MDA, GSH/GSSG, and SOD levels (Figure 5(c)) and reduced ROS production (Figure 5(d)).

### Resveratrol modulates the Keap1/Nrf2 axis through miR-200a

**Fig. 6:**
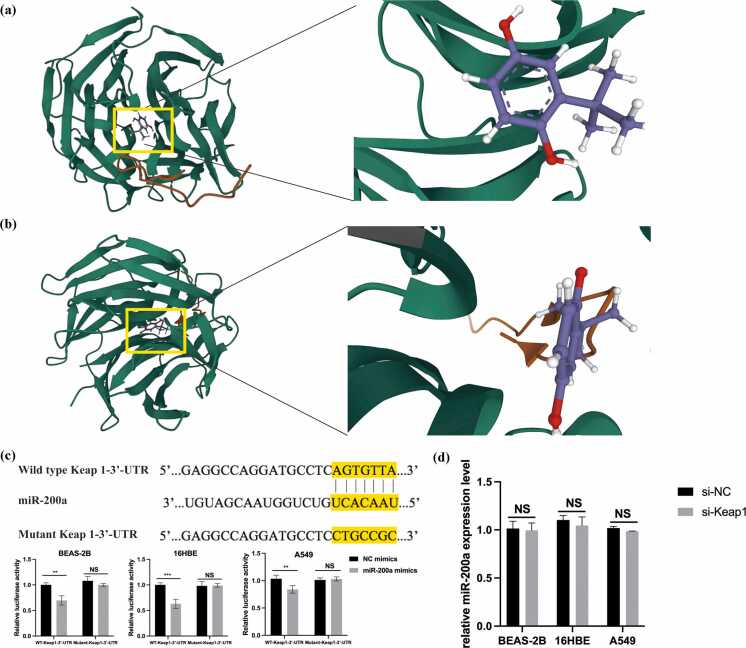
Proposed docking mode of RES in the binding site of Keap1 (PDB code 5FNQ). (a) Front view, and (b) top view of the docking mode of RES in the binding site of Keap1. (c) Dual-luciferase reporter assay of miR-200a with 3′-UTR vectors (wild type or mutant) of human Keap1 in BEAS-2B, 16HBE, and A549 cells. (d) Quantitative reverse transcription-polymerase chain reaction analysis of miR-200a expression in si-Keap1 transfected cells incubated with 5%CSE treatment. Abbreviations used: RES, resveratrol.

We explored the potential mechanism of resveratrol. According to molecular docking analysis, resveratrol was predicted to bind to Keap1’s binding site, potentially disrupting its interaction with Nrf2. The binding mode of resveratrol at the Keap1 binding site (PDB ID: 5FNQ) is illustrated in Figure 6(a) (front view) and Figure 6(b) (top view). Additionally, bioinformatics analysis predicted that miR-200a binds to the 3′-UTR of Keap1 mRNA. We performed a dual-luciferase reporter assay to confirm that miR-200a could directly bind to Keap1 3′-UTR. The wild-type 3′-UTR of Keap1 and its mutant 3′-UTR target sequences were cloned into luciferase reporter vectors and transfected into human bronchial epithelial cells along with miR-200a mimics. The results demonstrate miR-200a mimics significantly reduced the activity of the wild-type 3′-UTR reporter gene but had no effect on the activity of the mutant Keap1 3′-UTR reporter gene. Meanwhile, the NC mimics of miR-200a had no impact on the activity of either the wild-type or mutant Keap1 3′-UTR reporter gene (Figure 6(c)). Moreover, transfection with si-Keap1 did not affect the expression of miR-200a in CSE-stimulated 16HBE, BEAS-2B, and A549 cells (Figure 6(d)). Our results suggest that resveratrol may disrupt the Keap1-Nrf2 protein-protein interaction, potentially activating Nrf2 through miR-200a.

### Resveratrol alleviates pulmonary oxidative damage and inflammation in CS-exposed mice

**Fig. 7:**
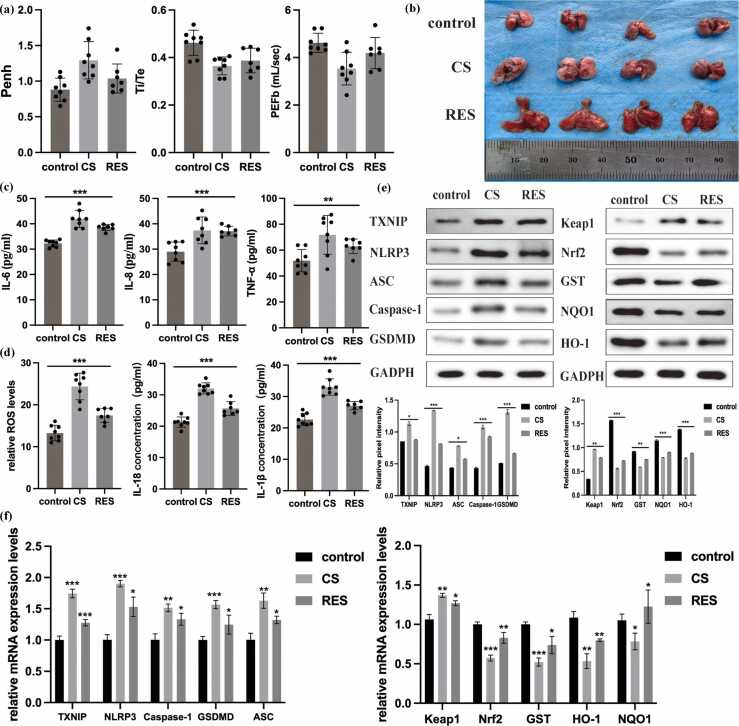
Resveratrol alleviated oxidative stress and Keap1/Nrf2 redox imbalance in CS-exposed mice. (a) Pulmonary function tests for all mice. (b) The congestion and edema of the lung tissue in mice of three groups. (c) The expression levels of IL-6, IL-8 and TNF-α in the serum of CS-exposed mice and mice with RES were significantly higher than those in the control group. (d) The oxidative stress levels in the BALF of CS and RES groups were significantly higher than that of the control group. (e) and (f) The protein and mRNA expression levels of Keap1/Nrf2 antioxidant pathway, TXNIP, NLRP3 and pyroptosis-related molecules in the lung tissues of different groups of mice. The data are expressed as the mean of at least 7 independent experiments±standard deviation, *P < 0.05, **P < 0.01 and ***P < 0.001 *versus* control group. Abbreviations used: CS, cigarette smoke; HBE, human bronchial epithelial; RES, resveratrol; BALF, bronchoalveolar lavage fluid.

A total of 50 C57BL/6 J mice were assigned to three groups: normal air exposure (control group), CS exposure, and resveratrol treatment during CS exposure (RES group). Due to the toxicity of prolonged CS exposure for six months, only 8 mice remained in the CS group and 7 in the RES group. Before we sacrificed all the mice, we also performed pulmonary function for all of the C57BL/6 J mice (Table 1). In a mouse model of CS exposure, CS exposure significantly impaired pulmonary function (Figure 7(a)), and caused histological damage including alveolar collapse and inflammatory infiltration (Figure 7(b)), and reduced the levels of inflammatory cytokines (Figure 7(c)). These changes were partially reversed in mice treated with resveratrol. Oxidative stress markers and inflammatory cytokines in bronchoalveolar lavage fluid were significantly reduced in the resveratrol-treated group (Figure 7(d)). Additionally, resveratrol restored Nrf2 and antioxidant gene expression levels, and reduced pyroptosis-related markers (Figure 7(e) and (f)). These results suggest that resveratrol mitigates oxidative stress and inflammation in vivo by restoring the Keap1/Nrf2 redox balance.

**Table 1:** Pulmonary function tests in the mice of different group.

	Min			25%			Median			75%			Max			95%CI					
Indexes	Control	CS	RES	Control	CS	RES	Control	CS	RES	Control	CS	RES	Control	CS	RES	Control		CS		RES	
																Lower	Upper	Lower	Upper	Lower	Upper
**Ti (ms)**	**0.0637**	**0.0553**	**0.05098**	**0.06509**	**0.05675**	**0.05628**	**0.06896**	**0.06141**	**0.0647**	**0.07156**	**0.06465**	**0.07859**	**0.07452**	**0.07512**	**0.09888**	**0.06559**	**0.07182**	**0.0571**	**0.06676**	**0.05541**	**0.08264**
**Te (ms)**	**0.1203**	**0.1259**	**0.1456**	**0.1341**	**0.1331**	**0.1568**	**0.1539**	**0.1617**	**0.1812**	**0.1664**	**0.1731**	**0.2436**	**0.1796**	**0.2381**	**0.2523**	**0.1334**	**0.1669**	**0.1362**	**0.1876**	**0.1564**	**0.2268**
Rpef	0.1431	0.2093	0.1011	0.15	0.2238	0.161	0.1692	0.2475	0.2039	0.2133	0.2736	0.2987	0.2191	0.3023	0.3018	0.1511	0.2018	0.2237	0.2745	0.1357	0.3202
EIP (ms)	2.351	2.276	2.279	2.446	2.434	2.33	2.61	2.668	2.449	2.973	2.932	2.952	3.226	3.01	3.534	2.445	2.948	2.438	2.889	2.314	2.948
EEP (ms)	34.37	34.66	55.92	51.74	46.8	75.26	58.06	66.35	80.71	68.37	78.12	112.3	84.37	154.2	161.5	44.93	73.06	44.9	91.35	66.03	120.6
TVb (mL)	0.2171	0.2103	0.169	0.2294	0.2153	0.2054	0.2388	0.2391	0.2153	0.2475	0.2535	0.226	0.2485	0.2652	0.2313	0.2278	0.2467	0.2196	0.2534	0.1892	0.2325
**Penh**	**0.6516**	**0.9908**	**0.7459**	**0.7425**	**1.083**	**0.78**	**0.8785**	**1.24**	**0.872**	**1.028**	**1.534**	**1.132**	**1.129**	**1.74**	**1.442**	**0.7398**	**1.013**	**1.08**	**1.483**	**0.7767**	**1.172**
PAU	1.344	1.084	1.154	1.367	1.191	1.251	1.533	1.274	1.345	1.638	1.338	1.581	1.863	1.435	1.858	1.391	1.67	1.172	1.356	1.206	1.596
EF50 (mL/s)	0.2415	0.1636	0.105	0.2421	0.1834	0.1601	0.2623	0.2296	0.186	0.2853	0.2543	0.2504	0.3159	0.3125	0.2668	0.2454	0.2872	0.1859	0.2636	0.1328	0.2506
Tr (ms)	0.05625	0.06439	0.06299	0.06043	0.06891	0.07657	0.06586	0.08313	0.09461	0.07413	0.09061	0.1144	0.07724	0.1172	0.1181	0.06032	0.0727	0.07009	0.09553	0.07404	0.1146
PIF (mL/s)	6.322	5.799	3.862	6.464	6.238	5.403	6.681	6.988	6.558	7.135	7.783	7.356	7.687	8.369	7.736	6.454	7.173	6.248	7.737	4.791	7.904
**PEF (mL/s)**	**4.18**	**3.286**	**2.418**	**4.333**	**3.413**	**3.074**	**4.482**	**4.159**	**3.566**	**5.083**	**4.374**	**4.25**	**5.233**	**5.362**	**4.648**	**4.296**	**4.932**	**3.506**	**4.596**	**2.843**	**4.327**
F (BPM)	311.4	304.8	224.6	318.9	314.1	309.3	331	332.9	329.5	364.6	342.7	397.8	376.5	394.9	400.2	319.3	358.3	313.6	356.4	270.2	405.7
MVb (mL)	78.3	63.87	42.62	79.93	69.2	62.03	83.83	80.54	70.53	86.22	93.88	82.1	96.6	107	88.6	79.81	88.73	69.47	92.77	52.79	87.3

Bold values indicate statistically significant differences between groups (*P* < 0.05).

Abbreviations used: RES, Resveratrol; CI, confidence interval; BPM, beats per minute; PIF, peak inspiratory flow; PAU, pause; EIP, end-inspiratory pause; EEP, end-expiratory pause.

## Discussion

CS is a major environmental risk factor contributing to the development and progression of COPD, especially among males, where smoking prevalence remains high.^21^ The ongoing burden of CS-related lung diseases highlights the urgent need to elucidate the underlying mechanisms and identify novel therapeutic strategies. In this study, we utilized a CSE-induced injury model in human bronchial epithelial cells to investigate interplay between oxidative stress, inflammation, and pyroptosis. We focused on the Keap1/Nrf2 antioxidant pathway and examined the potential protective effects of resveratrol, a naturally occurring polyphenol.

Oxidative stress, resulting from an imbalance between ROS production and antioxidant defenses, is a well-established hallmark of COPD pathogenesis.^22^ Sustained oxidative stress promotes inflammatory responses, which play a central role in disease progression.^23^ The thioredoxin (Trx) system, which maintains redox homeostasis through disulfide reductase activity, is negatively regulated by thioredoxin-interacting protein (TXNIP). TXNIP inhibits Trx function and contributes to oxidative stress by binding to its active site.^24^, ^25^ Moreover, under elevated ROS conditions, TXNIP has been shown to dissociate from Trx and interact with the NLRP3 inflammasome, promoting its activation and driving pyroptosis and inflammation.^26^ Consistent with these findings, we observed that TXNIP expression was upregulated in CSE-treated human bronchial epithelial cells. Silencing TXNIP using siRNA significantly suppressed the expression of NLRP3, caspase-1, ASC, and gasdermin D, as well as reduced the release of IL-1β and IL-18. These results implicate TXNIP as a key mediator of inflammasome-dependent pyroptosis in this model. However, TXNIP knockdown did not significantly affect ROS levels in our model, implying that TXNIP functions downstream of ROS in this context. These results support the notion that CSE-induced pyroptosis is, at least in part, mediated through TXNIP-NLRP3 signaling pathway. This process is crucial for maintaining the basal state of the cell, ensuring that the antioxidant response machinery is not over-activated in the absence of stress.

The Keap1/Nrf2 pathway is a pivotal defense mechanisms against oxidative injury. Under homeostatic conditions, Nrf2 is sequestered in the cytoplasm by Keap1and targeted for proteasomal degradation. This process is crucial for maintaining the basal state of the cell, ensuring that the antioxidant response machinery is not over-activated in the absence of stress. Upon oxidative stress, Nrf2 dissociates from Keap1, translocates to the nucleus, and activates transcription of antioxidant genes, including HO-1, NQO1, and GST.^27^ A published paper pointed out that the Nrf2-antioxidant response element signaling pathway plays a major role in the cellular defense against oxidative stress. It controls the expression of genes whose protein products are involved in the detoxication and elimination of reactive oxidants through conjugative reactions and by enhancing cellular antioxidant capacity.^28^ Similarly, Ohtsuji *et al.*^29^ emphasized the significance of Nrf2 in activating antioxidant response element dependent genes, highlighting its potential role in the cell’s antioxidant defense mechanism. In our study, CSE exposure upregulated Keap1 and downregulated Nrf2 and its target genes in both human bronchial epithelial cells and mouse lung tissues. Pharmacological Nrf2 activation using tBHQ or Keap1 knockdown restored antioxidant gene expression, reduced ROS production, and decreased TXNIP expression, indicating that Keap1 functions upstream of Nrf2 in the oxidative stress response. We also investigated the regulatory role of microRNA-200a (miR-200a), a microRNA known to target Keap1 mRNA. Prior studies have shown that miR-200a expression is reduced in oxidative conditions, leading to increased Keap1 levels and impaired antioxidant defense.^30^ Researchers demonstrated that miR-200a silencing was observed in breast cancer cells, and re-expression of miR-200a targeted the Keap1 3′-UTR, leading to Keap1 mRNA degradation. Loss of this regulatory mechanism may contribute to the dysregulation of Nrf2 activity.^31^ Our data confirmed that CSE suppressed miR-200a expression, which coincided with increased Keap1 and decreased Nrf2 signaling. These findings suggest that miR-200a may serve as a key modulator of the Keap1/Nrf2 axis under CSE treatment, contributing to pyroptosis and inflammation. Additionally, existing research showed that miR-200a inhibited 3′-UTR activity of Keap1 in bone marrow mesenchymal stem cells (BMSCs). Transplantation of BMSCs with overexpression of miR-200a increased locomotor function recovery of rats, while decreasing MDA level and increasing SOD, and Nrf2 expression together with its downstream protein expressions, further emphasizing the role of miR-200a in regulating the Keap1/Nrf2 pathway.^32^

Resveratrol, a naturally occurring polyphenol found in grapes and other plants, has demonstrated antioxidant and anti-inflammatory properties in various disease models.^33^, ^34^ Resveratrol also activates Nrf2 and glutamate-cysteine ligase in human bronchial epithelial cells to reverse CS-induced reduction of GSH levels, which play a role in the treatment of COPD antioxidative effect.^35^ In the current study, resveratrol treatment significantly improved antioxidant capacity, inhibited TXNIP and inflammasome activation, and attenuated CSE-induced pyroptosis and inflammatory cytokines release. These protective effects were associated with enhanced miR-200a expression, reduced Keap1 levels, and reactivation of Nrf2-dependent antioxidant responses. It is important to note that while our findings highlight the potential of resveratrol as a modulator of oxidative stress and pyroptosis, the degree of rescue was partial-in some assays, resveratrol failed to restore readouts to control levels. Therefore, we caution against overinterpreting the extent of its protective effects. Moreover, although our *in vitro* CSE model mimics certain aspects of COPD pathogenesis, it does not fully recapitulate the complexity of the human disease. Consequently, we cannot claim direct therapeutic efficacy of resveratrol in COPD without further validation in appropriate in vivo models.

Finally, while we primarily focused on the Keap1/Nrf2 pathway, resveratrol may also exert its effects through alternative signaling cascades. Resveratrol is a polyphenolic compound that has been extensively studied for its diverse biological activities, including antioxidant, anti-inflammatory, and anti-carcinogenic properties.^36^ For example, prior reports implicate the Syk/NF-κB and SIRT1/PGC-1α pathways in resveratrol-mediated protection against airway inflammation. The precise interplay among these pathways warrants further investigation. Additionally, structural optimization of resveratrol and development of derivatives with improved bioavailability may represent promising future directions. Finally, while we primarily focused on the Keap1/Nrf2 pathway, resveratrol may also exert its effects through alternative signaling cascades.

## Conclusion

The results suggest that CSE-induced oxidative stress leads to NLRP3 inflammasome activation and pyroptosis in human bronchial epithelial cells. Disruption of the Nrf2 pathway by CSE exacerbates oxidative damage, while resveratrol, through its activation of the Nrf2 pathway, mitigates oxidative stress and prevents pyroptosis. These findings highlight the potential of resveratrol as a therapeutic agent for protecting lung epithelial cells from smoke-induced injury through Nrf2 activation.

## Funding and support

This work was supported by grants from the 10.13039/501100007129Shandong Provincial Natural Science Foundation (No. ZR2023QH397) and the 10.13039/501100001809National Natural Science Foundation of China (No. 82173173).

## Author contributions

M.Z. collected, organized the data, and wrote the most manuscript. C.H. helped analyze the data. G.Y. helped with the statistical design. Y.H. contributed to the cell culture and identification. Y.Q. developed the concept, supervised the project, conceived the experiments, and revised the manuscript.

## CRediT authorship contribution statement

**Yiqing Qu:** Writing – review & editing, Supervision, Funding acquisition, Conceptualization. **Yajie Hu:** Validation, Investigation. **Guang Yang:** Visualization, Formal analysis. **Chenyang Hu:** Validation, Investigation, Formal analysis. **Mengyu Zhang:** Writing – original draft, Visualization, Validation, Methodology, Funding acquisition, Formal analysis, Data curation, Conceptualization.

## Declarations of interest

The authors declare that they have no competing interests.

## Data Availability

Data will be made available on request.
